# Supplementary material for: Testing for interactions in multivariate data
Source: PLoS One. 2026 Nov 10;21(11):e0338580. doi: 10.1371/journal.pone.0338580 (PMC13750587; doi:10.1371/journal.pone.0338580)
Supplement: Supplementary file 1 [file pone.0338580.s001.pdf]

Testing for Interactions in Multivariate Data: Appendix

W Penny, T Sambrook and L Renoult

School of Psychology,
University East Anglia, UK.

June 25, 2026

1 Distributions over Matrices

The following definitions and properties are taken from [1].

1.1 Normal distributions

The matrix-normal distribution over the $[d_1 \times d_2]$ matrix X is

$$p(X) = \mathsf{N}_{d_1,d_2}(X; M, V, \Sigma) \tag{1}$$

with M being the mean, V being the $[d_1 \times d_1]$ covariance over rows, and Σ being the $[d_2 \times d_2]$ covariance over columns. It is equivalent to the multivariate normal

$$\begin{aligned} p(x_v) &= \mathsf{N}_d(x_v; m_v, \Sigma \otimes V) \\ x_v &= \mathsf{vec}(X) \\ m_v &= \mathsf{vec}(M) \\ d &= d_1 d_2 \end{aligned} \tag{2}$$

where $\otimes$ denotes the Kronecker product, $\mathsf{x} = \mathsf{vec}(\mathsf{X})$ vectorises X column by column, and $\mathsf{N}_d(x; m, C)$ is a multivariate normal distribution over d-dimensional random vector x with mean m and covariance C.

1.2 Wishart distribution

The inverse-Wishart distribution over the $[p \times p]$ matrix Σ is given by

$$\begin{aligned} p(\Sigma) &= \mathsf{IW}_p(\Sigma; \Psi, \alpha) \\ \bar{\Sigma} &= \frac{\Psi}{\alpha - p - 1} \end{aligned} \tag{3}$$

with mean $\bar{\Sigma}$ if $\alpha > p + 1$. Its equivalent to stating the Σ^{-1} follows a Wishart distribution. We have

$$\begin{aligned} \mathsf{IW}_p(\Sigma; \Psi, \alpha) &= \frac{1}{Z} \exp\left(-\frac{1}{2}\mathsf{Tr}[\Sigma^{-1}\Psi]\right) \\ Z &= \frac{\Gamma_p[\eta/2]|\Sigma|^{\alpha/2}}{2^{-\eta p/2}|\Psi|^{\eta/2}} \\ \eta &= \alpha - p - 1 \end{aligned} \tag{4}$$

1.3 Matrix T-distribution

The matrix-T distribution over the $[d1 \times d2]$ matrix X is

$$p(X) = T_{d_1,d_2}(X; M, V, \Psi, \alpha) \tag{5}$$

with mean M (if $\alpha > 1$ otherwise undefined), $[d_1 \times d_1]$ scale matrix V, $[d_2 \times d_2]$ scale matrix Ψ, and degrees of freedom α. We have

$$\begin{aligned} T_{p,m}(X; M, V, \Psi, \alpha) &= \frac{1}{Z}|I_p + V^{-1}(X - M)\Psi^{-1}(X - M)^T|^{-0.5\eta} \\ Z &= |V|^{-0.5m}|\Psi|^{-0.5p}\frac{\Gamma_p[0.5(\alpha + p - 1)]\pi^{0.5mp}}{\Gamma_p[0.5\eta]} \\ \eta &= \alpha + m + p - 1 \end{aligned} \tag{6}$$

Note that evaluating this density involves computing the inverses V^{-1} and Ψ^{-1}.

If $C = AXB$ where A is a $[c_1 \times d_1]$ row contrast matrix and B is a $[d_2 \times c_2]$ column contrast matrix then, according to theorem 4.3.8 in [1], we have

$$p(C) = T_{c_1,c_2}(C; AMB, AVA^T, B^T\Psi B, \alpha) \tag{7}$$

According to theorem 4.3.3 in [1] we have

$$p(X^T) = T_{d_2,d_1}(X^T; M^T, \Psi, V, \alpha) \tag{8}$$

Another property (theorem 4.3.1) is that

$$\mathsf{Cov}(\mathsf{Vec}(X^T)) = \frac{V \otimes \Psi}{\alpha - 2} \tag{9}$$

for $\alpha > 2$. So we should therefore have

$$\mathsf{Cov}(\mathsf{Vec}(X)) = \frac{\Psi \otimes V}{\alpha - 2} \tag{10}$$

This then matches up with the equivalent formula for the matrix normal.

1.4 Multivariate T-distribution

The multivariate T distribution $\mathsf{T}_d(x; m, \Sigma, \alpha)$ over d-dimensional random vector x has mean m, scale matrix Σ, degrees of freedom α, and covariance

$$\mathsf{Cov}(x) = \frac{\alpha}{\alpha - 2}\Sigma \tag{11}$$

Given $[d \times r]$ contrast matrix C we have

$$\begin{aligned} c &= C^T x \\ p(c) &= \mathsf{T}_r(c; C^T m, C^T \Sigma C, \alpha) \end{aligned} \tag{12}$$

2 Model Evidence and Bayes Factors

The model evidence for the multivariate linear model with conjugate priors (see main text) is

$$\begin{aligned} p(Y|X) &= \int\int P(Y|X,B,\Sigma)p(B|\Sigma)p(\Sigma)dBd\Sigma \quad (13) \\ &= \frac{|V_n|^{M/2}}{|V_0|^{M/2}}\frac{|\Psi_0|^{\alpha_0/2}}{|\Psi_n|^{\alpha_n/2}}\frac{\Gamma_M(\alpha_n/2)}{\Gamma_M(\alpha_0/2)}\pi^{-MN/2} \end{aligned}$$

where $|V_n|$ is the determinant of V_n and Γ_M is the generalised gamma function [1]. A derivation is provided in section 3.4 of Ramirez-Hassan's book [2] which is available digitally at `https://bookdown.org/aramir21/IntroductionBayesianEconometricsGuidedTour/`.

The Bayes factor is therefore

$$\frac{p(Y|X^{alt})}{p(Y|X^{null})} = \left(\frac{|V_n^{alt}|}{|V_n^{null}|}\frac{|V_0^{null}|}{|V_0^{alt}|}\right)^{M/2}\left(\frac{|\Psi_n^{null}|}{|\Psi_n^{alt}|}\right)^{\alpha_n/2} \quad (14)$$

where we have assumed that $\Psi_0^{alt} = \Psi_0^{null}$ (this is the assumption for our application to EEG data but can be relaxed for other applications). One way to understand the second term is to write it as

$$\left(\frac{|\Psi_n^{null}|}{|\Psi_n^{alt}|}\right)^{\alpha_n/2} = \left(\frac{\prod_{m=1}^{M}\lambda_m^{null}}{\prod_{m=1}^{M}\lambda_m^{alt}}\right)^{\alpha_n/2} \quad (15)$$

where λ_m^{null} and λ_m^{alt} are the eigenvalues of Ψ_n^{null} and Ψ_n^{alt} respectively. These correspond to error variances in the mth eigen-direction. The product of these eigenvalues corresponds to the error volume in M-dimensional space (see Fig 2.7 in [3] for an illustration). The alternative model is to be preferred if it has a smaller error volume. The log Bayes factor in favour of the alternative versus null model is then

$$\begin{aligned} \log BF &= \frac{\alpha_n}{2}\sum_{m=1}^{M}\log\frac{\lambda_m^{null}}{\lambda_m^{alt}} \quad (16) \\ &- \frac{M}{2}\left[\log\frac{|V_0^{alt}|}{|V_n^{alt}|} - \log\frac{|V_0^{null}|}{|V_n^{null}|}\right] \end{aligned}$$

3 Predictive Density

The following property is not made use of in the current paper but may be useful in future applications. Given N_0 new observations Y_0 the predictive density is

$$\begin{aligned} p(Y_0|Y) &= T_{N_0,M}(Y_0;\hat{Y}_0,\hat{V}_n,\Psi_n,\hat{\alpha}) \quad (17) \\ \hat{Y}_0 &= X_0B_n \\ \hat{V}_n &= I_{N_0} + X_0V_nX_0^T \\ \hat{\alpha} &= \alpha_n - M + 1 \end{aligned}$$

and Ψ_n is defined in the main text under "Posterior Density". The predictive density could be used as part of a Bayesian inference approach to infer diagnostic labels from clinical imaging data

where Y is the training data, Y_0 are the new data to label, $p(C)$ is a prior distribution over labels, and the posterior distribution over labels is

$$p(C|Y_0, Y) = \frac{p(Y_0|Y, C)p(C)}{\sum_k p(Y_0|Y, C = k)p(C = k)} \tag{18}$$

and $p(Y_0|Y, C)$ takes the form of Eq 17 with parameters $(\alpha_n, B_n, V_n, \Psi_n)$ specific to label C.

References

[1] A Gupta and D Nagar. *Matrix Variate Distributions.* Chapman and Hall, 2000.

[2] A Ramirez-Hassan. *Introduction to Bayesian Data Modeling: A Guided toolkit using R.* In Development, 2025.

[3] C.M. Bishop. *Pattern Recognition and Machine Learning.* Springer, New York, 2006.
